# Molecular characterization of invasive capsule null *Neisseria meningitidis* in South Africa

**DOI:** 10.1186/s12866-017-0942-5

**Published:** 2017-02-21

**Authors:** Karistha Ganesh, Mushal Allam, Nicole Wolter, Holly B. Bratcher, Odile B. Harrison, Jay Lucidarme, Ray Borrow, Linda de Gouveia, Susan Meiring, Monica Birkhead, Martin C. J. Maiden, Anne von Gottberg, Mignon du Plessis

**Affiliations:** 1 National Institute for Communicable Diseases (NICD), A division of the National Health Laboratory Service (NHLS), Johannesburg, South Africa; 2 School of Pathology, Faculty of Health Sciences, University of the Witwatersrand, Johannesburg, South Africa; 3 Department of Zoology, University of Oxford, Oxford, UK; 4 Meningococcal Reference Unit, Public Health England, Manchester Medical Microbiology Partnership, Manchester Royal Infirmary, Manchester, UK

**Keywords:** Capsule null locus, Invasive disease, *Neisseria meningitidis*, Africa, ST-53, ST-192

## Abstract

**Background:**

The meningococcal capsule is an important virulence determinant. Unencapsulated meningococci lacking capsule biosynthesis genes and containing the capsule null locus (*cnl*) are predominantly non-pathogenic. Rare cases of invasive meningococcal disease caused by *cnl* isolates belonging to sequence types (ST) and clonal complexes (cc) ST-845 (cc845), ST-198 (cc198), ST-192 (cc192) and ST-53 (cc53) have been documented. The clinical significance of these isolates however remains unclear. We identified four invasive *cnl* meningococci through laboratory-based surveillance in South Africa from 2003 through 2013, which we aimed to characterize using whole genome data.

**Results:**

One isolate [NG: P1.7-2,30: F1-2: ST-53 (cc53)] contained *cnl* allele 12, and caused empyema in an adult male with bronchiectasis from tuberculosis, diabetes mellitus and a smoking history. Three isolates were NG: P1.18-11,42-2: FΔ: ST-192 (cc192) and contained *cnl* allele 2. One patient was an adolescent male with meningitis. The remaining two isolates were from recurrent disease episodes (8 months apart) in a male child with deficiency of the sixth complement component, and with the exception of two single nucleotide polymorphisms, contained identical core genomes. The ST-53 (cc53) isolate possessed alleles for NHBA peptide 191 and fHbp variant 2; whilst the ST-192 (cc192) isolates contained NHBA peptide 704 and fHbp variant 3. All four isolates lacked *nadA.* Comparison of the South African genomes to 61 additional *cnl* genomes on the PubMLST *Neisseria* database (http://pubmlst.org/neisseria/), determined that most putative virulence genes could be found in both invasive and carriage phenotypes.

**Conclusions:**

Although rare, invasive disease by *cnl* meningococci may be associated with host immunodeficiency and such patients may benefit from protein-based meningococcal vaccines.

**Electronic supplementary material:**

The online version of this article (doi:10.1186/s12866-017-0942-5) contains supplementary material, which is available to authorized users.

## Background

*Neisseria meningitidis* (meningococcus) is a commensal of the human upper respiratory tract, which occasionally causes meningitis and sepsis, particularly in infants and young adults. Meningococci may express one of twelve antigenically distinct capsules, however, invasive disease is mostly caused by serogroups A, B, C, W, X or Y. The polysaccharide capsule is an important virulence determinant and aids in evading the host immune response [1]. Enzymes for capsule biosynthesis and transport are encoded by a single cluster of genes termed the capsular polysaccharide synthesis (*cps*) locus, which is divided into six regions arranged in the order of D-A-C-E-D’-B [2]. Polysaccharide synthesis is encoded by region A genes which vary according to serogroup, whilst regions B (*ctrE*-*F*) and C (*ctrA*-*D*) are responsible for capsular transport [3]. Region D (*rfbA-C* and *galE*) is involved in lipooligosaccharide biosynthesis and D’ (*rfbA2-C2* and *galE2*), a truncated copy of region D, is non-functional [4]. Region E is not involved in polysaccharide synthesis or transport, but is hypothesized to regulate these processes [5].

Loss of capsule expression may be caused by horizontal genetic exchange, slipped-strand mispairing, point mutations or gene deletion in the *cps* locus [6, 7]. Meningococci which lack *cps* genes are regarded as non-pathogenic. However, rare cases of invasive disease caused by meningococci lacking regions A, B and C of the *cps* locus and containing a capsule null locus (*cnl*), have been reported in Germany, Canada, Burkina Faso and China [8–12]. Twenty-six *cnl* alleles (113-368bp) were defined in the PubMLST *Neisseria* spp. database (http://pubmlst.org/neisseria/) at the time of this study, with some alleles present in other *Neisseria* including *N. lactamica* and *N. gonorrhoeae* [6]. Genetic lineages described for invasive *cnl* isolates include those belonging to sequence type (ST) ST-192 (clonal complex (cc) 192), ST-198 (cc198) and ST-845 (cc845) [8, 9, 11, 12]. Clonal complex 53 isolates are typically associated with carriage [6, 13, 14] although invasive isolates have been documented on the PubMLST *Neisseria* database. Polysaccharide-based vaccines that target serogroups A, C, W, and Y are ineffective against *cnl* strains however, protein-based meningococcal vaccines developed for serogroup B, such as Bexsero® and the bivalent factor H-binding protein (fHbp) vaccine Trumenba®, have the potential to target non-serogroup B including *cnl* meningococci [15–17].

Through national laboratory-based surveillance for invasive meningococcal disease (IMD) from 2003 through 2013, we identified four *cnl* meningococci. We aimed to describe the respective cases and characterize the isolates using whole genome data.

## Results

### Identification and characterization of invasive capsule null meningococci

**Fig. 1:**
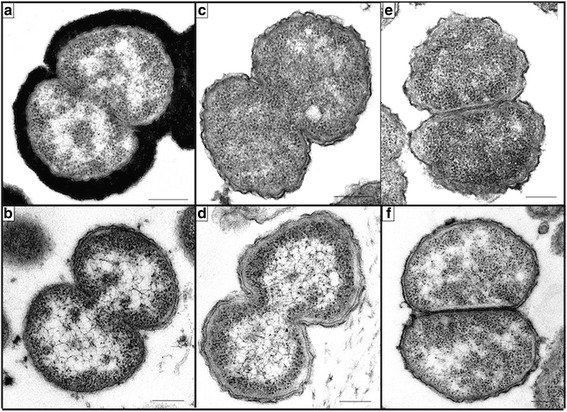
Transmission electron micrographs showing the presence of surface capsular polysaccharide for (**a**) *Neisseria meningitidis* serogroup W (ATCC 35559) and (**b**) absence of capsule for *Neisseria lactamica* (ATCC 23970). Clinical isolates from South Africa are depicted in (**c**) 29312 (**d**) 29306 (**e**) 41961 and (**f**) 41860. The scale bar represent 200 nm

From 2003 through 2013, 4770 cases of IMD were reported, with viable isolates available for 2988 (63%) cases. We identified five *N. meningitidis* isolates that were phenotypically and genotypically negative for serogroups A, B, C, W, X and Y. The isolates were also *ctrA* PCR negative but were *sodC* PCR positive. Transmission electron microscopy (TEM) confirmed the absence of a polysaccharide capsule (Fig. 1).

One of the five isolates, 37616, did not contain a *cnl* allele, belonged to ST-11147 (cc41/44) and contained capsule transport genes in region B (*ctrE* and *ctrF*) and lipopolysaccharide synthesis genes (*rfbA-C* and *galE*) in region D of the *cps* locus. All region A genes except for *ctrG* were absent, as well as *ctrA* from region C. The remaining four *ctrA* negative isolates lacked genes in regions A and C (*ctrA-D*) and contained a 114bp *cnl* allele. Further, these isolates contained all region D lipopolysaccharide synthesis genes and lacked region B genes. The locus was identified as *cnl* allele 12 in isolate 29312, and *cnl* allele 2 in isolates 29306, 41860 and 41961 (Additional file 1: Figure S1) [6]. Isolates 29306, 41860 and 41961 lacked the *fetA* locus. The finetypes for the four *cnl* isolates were as follows: isolate 29312 was NG: P1.7-2,30: F1-2: ST-53 (cc53), and isolates 29306, 41860 and 41961 were NG: P1.18-11,42-2: FΔ: ST-192 (cc192).

### Clinical case descriptions

The four invasive *cnl* meningococi were isolated from three patients, including one patient with recurrent meningococcal disease (Table 1). All three patients responded well to antibiotic therapy and subsequently recovered from their IMD episodes. The four *cnl* isolates were susceptible to all antimicrobials except for trimethoprim-sulfamethoxazole. Additional information regarding vaccination status and long-term complications for all three patients was sought, but unfortunately these data were not available.

**Table 1:** Patient demographic information and phenotypic and genotypic characteristics of four invasive capsule null *Neisseria meningitidis* isolates identified through national laboratory-based surveillance in South Africa, 2003–2013

Characteristic				
Patient	1	2	3 (Episode 1)^a^	3 (Episode 2)^a^
Gender	Male	Male	Male	Male
Age category (years)	45–64	15–24	5–9	5–9
HIV status	Negative	Unknown	Negative	Negative
Antiretroviral use	Not applicable	Unknown	Not applicable	Not applicable
Underlying disease	Diabetes mellitus, COPD	Unknown	C6 deficiency	C6 deficiency
Year of disease presentation	2006	2010	2011	2012
Province	Western Cape	Gauteng	Free State	Free State
Patient outcome	Recovered	Recovered	Recovered	Recovered
Specimen type	Pleural aspirate	CSF	CSF	Blood
Isolate				
Minimum Inhibitory Concentrations (μg/ml)				
Penicillin G	0.032 (S)	0.064 (S)	0.064 (S)	0.047 (S)
Ceftriaxone	≤0.002 (S)	≤0.002 (S)	≤0.002 (S)	≤0.002 (S)
Trimethoprim-sulfamethoxazole	8 (R)	12 (R)	3 (R)	3.8 (R)
Chloramphenicol	0.75 (S)	1 (S)	1 (S)	0.38 (S)
Rifampicin	0.008 (S)	0.032 (S)	0.064 (S)	0.032 (S)
Ciprofloxacin	0.008 (S)	0.008 (S)	0.006 (S)	0.006 (S)
Molecular characterization				
*cnl* allele (NEIS2743)	12	2	2	2
Strain designation	NG: P1.7-2,30: F1-2: ST-53 (cc53)	NG: P1.18-11, 42-2: FΔ: ST-192 (cc192)	NG: P1.18-11, 42-2: FΔ: ST-192 (cc192)	NG: P1.18-11, 42-2: FΔ: ST-192 (cc192)
Genome information				
Approx genome size (bp)	2,104,685	2,040,849	1,995,940	2,003,633
No. contigs	119	111	447	489
*Neisseria* PubMLST.org identification number	29312	29306	41961	41860

*Abbreviations*: *COPD* chronic obstructive pulmonary disease, *C6* sixth complement component, *CSF* cerebrospinal fluid, *S* susceptible, *R* resistant, *NG* non-groupable, *P1* PorA, *F* FetA, *ST* sequence type, *cc* clonal complex, *Δ* gene deletion

^a^Patient three presented with two episodes of invasive meningococcal disease in 2011 (episode 1) and 2012 (episode 2, 8 months later), respectively. He was diagnosed with deficiency of the sixth complement component (C6)

The first patient, an adult male, was previously diagnosed with multiple chronic illnesses including diabetes mellitus, hypertension, osteoarthritis and chronic obstructive pulmonary disease. He was a smoker, morbidly obese and had a right lower lobe lobectomy in 2003 due to damage from a previous tuberculosis infection. In 2006, he was diagnosed with empyema and *N. meningitidis* was cultured from the pleural fluid (isolate 29312). The second patient, an adolescent male, was diagnosed with meningococcal meningitis in 2010 (isolate 29306). Unfortunately, information regarding underlying disease conditions and the severity of disease could not be obtained. The third patient was a male child with deficiency of the sixth complement component (C6). In 2011, he was diagnosed with meningitis (isolate 41961), however, 8 months later, in 2012, he presented with fever and disorientation and *N. meningitidis* was isolated from the blood (isolate 41860). He was prescribed life-long treatment with penicillin. His mother received a dose of the quadrivalent conjugate vaccine (Menactra®) in 2015.

### Comparison of South African and other capsule null meningococcal genomes

**Fig. 2:**
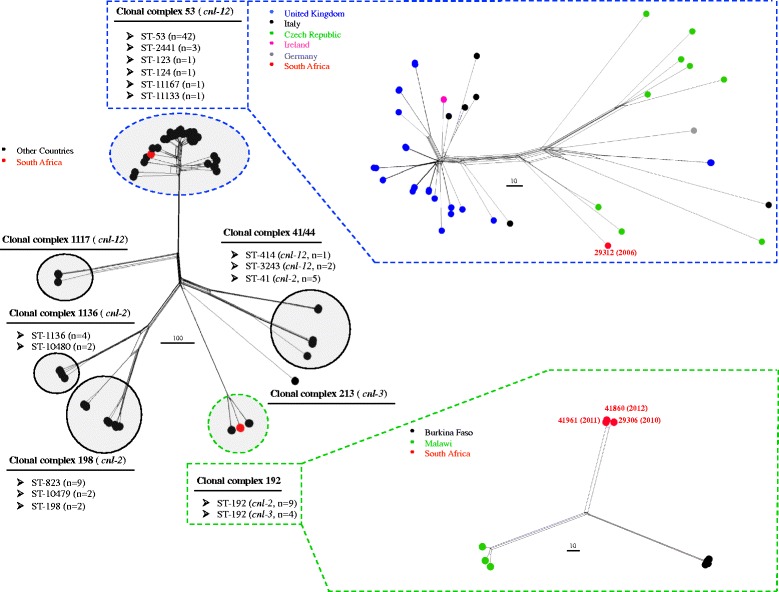
Phylogenetic analysis of 959 of 1605 core genes (cgMLST) genes in capsule null *Neisseria meningitidis* isolates (*n* = 93) belonging to clonal complexes (cc) 53 (*n* = 49), cc198 (*n* = 13), cc192 (*n* = 13), cc1136 (*n* = 6), cc41/44 (*n* = 8), cc1117 (*n* = 3) and cc213 (*n* = 1). Individual cgMLST phylogenies are also illustrated for cc53 and cc192. Clusters are highlighted in grey and the invasive South African isolates are represented by red nodes. Isolates 41860 and 41961 were from recurrent invasive disease episodes in the same patient. The scale bars represent the number of variant loci. All genomes are available on the http://pubmlst.org/neisseria website

The four South African (SA) isolates were compared to 89 *cnl* meningococcal genomes available on the PubMLST *Neisseria* database, including four cc192 Burkina Faso isolates that were sequenced as part of this study (Additional file 2: Table S1). The remaining 85 isolates belonged to seven clonal complexes including cc53 (*n* = 48), cc198 (*n* = 13), cc41/44 (*n* = 8), cc1136 (*n* = 6), cc192 (*n* = 6), cc1117 (*n* = 3) and cc213 (*n* = 1). All *cnl* meningococci (*n* = 93) harboured *fHbp* and *nhba*, but lacked *nadA*. Phylogenetic analysis of 53 ribosomal MLST (rMLST) loci clustered the 93 *cnl* isolates by clonal complex (data not shown). Data for 646 of 1605 core genome MLST (cgMLST) loci were incomplete and were excluded from further analysis. Phylogenetic analysis of the remaining 959 of 1605 cgMLST loci in all *cnl* isolates (*n* = 93), demonstrated clustering by clonal complex (Fig. 2). Isolates within each respective clonal complex contained identical *cnl* alleles, except for cc41/44 which contained alleles 2 or 12; and cc192 which contained alleles 2 or 3. Additional analysis of 117 putative virulence loci in 51 carriage and 14 invasive *cnl* isolates including those from South Africa, determined no mutually exclusive loci or alleles (data not shown). Most putative virulence loci were identified in both carriage and invasive isolates (97/117), and the remaining 20 loci were absent in all 65 isolates.

### Clonal complex 53

At the time of analysis, in addition to isolate 29312, complete genome data were available for 48 non-groupable cc53 isolates in the PubMLST *Neisseria* database. Forty-seven were carriage isolates from either the UK (*n* = 31), Czech Republic (*n* = 9), Italy (*n* = 6) or Germany (*n* = 1), and one was an invasive isolate from Ireland in 2012. The cc53 isolates (*n* = 49) belonged to one of six STs, namely, ST-53 (*n* = 42), ST-2441 (*n* = 3), ST-123 (*n* = 1), ST-124 (*n* = 1), ST-11167 (*n* = 1) or ST-11133 (*n* = 1). All cc53 isolates harboured *cnl* allele 12 flanked by *galE* allele 16, and one of five *tex* alleles (5, 787, 222, 788 or 969). *GalE* allele 16 was present in one cc41/44 isolate, also harbouring *cnl* allele 12. All cc53 isolates possessed alleles 24 or 102 for the fHbp family 2/subfamily A antigen. The NHBA allele 65 (peptide 58) was present in all of the cc53 isolates, except the SA isolate which harboured allele 149 (peptide 191).

Using seven-locus MLST, the cc53 isolates were resolved into six clusters (data not shown). Ribosomal MLST further resolved these isolates into 21 clusters and the SA isolate had a unique rMLST profile (data not shown). Core genome MLST indicated that the SA isolate was more closely related to two carriage isolates circulating in the Czech Republic in 1993, than to carriage isolates from the UK, Italy and Germany, and the invasive isolate from Ireland (Fig. 2). Overall, 221/959 (23%) cgMLST loci had identical nucleotide sequences amongst the cc53 isolates (*n* = 49).

### Clonal complex 192

Genome data were available in the PubMLST *Neisseria* database for four Burkina Faso isolates sequenced as part of this study, and six carriage isolates from Malawi. Analysis of the *cps* locus of the Burkina Faso isolates confirmed the absence of regions A, B and C and the presence of *cnl* allele 3 flanked by *galE* allele 365 and *tex* allele 826 [8]. In contrast, the SA and Malawi isolates harboured *cnl* allele 2 flanked by *galE* allele 161 and *tex* allele 235. All *galE* and *tex* alleles were unique to cc192 *cnl* isolates, and were not found in any other clonal complex nor in encapsulated isolates in the PubMLST *Neisseria* database. The Burkina Faso isolates were finetype NG: P1.18-11,42: FΔ: ST-192 (cc192) whilst the SA and Malawi isolates were NG: P1.18-11,42-2: FΔ: ST-192 (cc192). All 13 isolates harboured allele 112 for the fHbp family 3/subfamily A antigen and lacked both the *fetA* and *nadA* loci. Eleven of 13 isolates had allele 621 for NHBA peptide 704.

Ribosomal MLST and cgMLST resolved the 13 cc192 isolates into three groups, which were congruent with country of origin. The SA and Malawi isolates were more closely related to each other (variable cgMLST loci: 183/959, 19%), compared to the Burkina Faso isolates, which differed from the SA meningococci by 201 (21%) loci and the Malawi meningococci by 217 (23%) loci, respectively (*p* = 0.06) (Fig. 2). Isolate 29306 from patient two, differed by 13 (1%) and 15 cgMLST loci (2%) from isolates 41961 and 41860 which were from recurrent IMD episodes in patient three, respectively. Isolates 41961 and 41860 differed from each other by two cgMLST metabolic loci (0.2%) (Additional file 3: Table S2). Further nucleotide analysis indicated that both genes differed by one nucleotide. Overall, 660/959 (69%) of cgMLST loci shared identical nucleotide sequences amongst the 13 cc192 isolates.

## Discussion

Four invasive *cnl* meningococci were detected from three patients, including recurrent IMD in a C6 complement deficient patient. There was no epidemiological link between patients, and one isolate was NG: P1.7-2,30: F1-2: ST-53 (cc53) whilst the remaining three were NG: P1.18-11,42-2: FΔ: ST-192 (cc192). These genotypes were different to invasive *cnl* isolates reported in other countries [8–12]. One invasive isolate did not contain a *cnl* allele*,* but lacked most genes from regions A and C of the *cps*. This was similar to that described previously in a non-groupable cc41/44 carriage isolate from the USA [7].

Meningococci of cc53 with *cnl* have previously been found in 7% of *N. meningitidis* isolates from healthy carriers in Germany [6, 13]. Meningococcal carriage data from the African meningitis belt suggest that cc53 is not common in this region [18–20] with searches in the PubMLST *Neisseria* database identifying only three other non-groupable cc53 carriage isolates from this region: two from Senegal and one in Ethiopia. In addition to an invasive Irish isolate which possessed the same finetype as the South African isolate, 11 other invasive non-groupable cc53 isolates were identified in Cuba, Cyprus, France and the UK, of which six isolates were NG: P1.7,30: F-ND: ST-53 (cc53). Carriage rates are currently unknown in South Africa and we have no knowledge of carried genotypes. Although we do not have genotypic data for all of our invasive isolates the earliest documentation of any cc53 strain in South Africa is the *cnl* isolate identified in 2006, and described in this study.

In contrast to cc53, cc192 has been reported among carriers in countries in the African meningitis belt including Burkina Faso [20], Ghana [19], The Gambia, Ethiopia, Mali, Uganda and Niger (PubMLST *Neisseria* database). According to the PubMLST *Neisseria* database, 95% (72/76) of cc192 isolates were isolated in Africa, and 4% (3/76) isolated in Europe; as opposed to carriage cc53 isolates which were predominantly isolated in Europe (74%, 312/419) and rarely observed in Africa (1%, 6/419). Furthermore, two cc192 carriage isolates were documented in Norway and Sweden, and one case of bacteremia was documented in France (PubMLST *Neisseria* database). The Swedish and French isolates shared the same strain designation as the South African and Malawi isolates, however genomic data were unavailable for these isolates. Three cases of invasive disease caused by *cnl* ST-192 isolates occurred in Burkina Faso in 2003 and 2004, two of which were included in our study along with two carriage isolates from 2003 [8, 20]. Core genome MLST analysis of 13 African isolates revealed three groups of cc192 isolates clustering according to their respective countries, however the dataset in this study was limited and more cc192 genomes would be required to validate this geographic clustering and fully describe the molecular epidemiology of this clone.

The lack of a polysaccharide capsule in disease-associated isolates implies that factors other than encapsulation may contribute to the ability of a strain to cause invasive disease, including underlying disease conditions of the host. Disease due to unencapsulated meningococci and recurrent IMD, have been described in immunocompromised patients who are deficient in terminal pathway complement proteins C5 through C9 [21–26]. In our study, one patient with C6 deficiency had recurrent IMD with the same *cnl* strain. A similar case was described in the USA in a 5-month-old male who was also diagnosed with C6 deficiency [22]. He was first diagnosed with meningitis followed by bacteremia six months later. Both episodes were likely to have been caused by the same unencapsulated meningococcal strain, based on the fact that the isolates were non-groupable by phenotypic serogrouping and had identical outer membrane vesicle profiles on SDS-PAGE, however additional genotypic data were not available for confirmation. These cases of recurrent IMD may suggest persistent carriage in close contacts which is further supported by Mueller et al. [20] who identified non-groupable cc192 meningococci at three consecutive monthly visits in six healthy carriers in Burkina Faso. In South Africa, chemoprophylaxis is recommended for close contacts of IMD patients to eradicate carriage, however this particular strain may have persisted within the family. This does not however exclude the possibility that the second isolate may have been re-acquired from an individual in the community. Philadelphia chromosome-positive (bcr-abl+) common acute lymphatic leukemia was reported in a patient with IMD caused by a ST-845 (cc845) *cnl* isolate in Germany, in 2004 [11]. In our study, the ST-53 (cc53) isolate was the only organism to be cultured from pleural fluid of a patient with empyema, indicating that this isolate was the most likely cause of invasive disease. Further, IMD by the ST-53 (cc53) isolate occurred in a patient who was immunocompromised due to diabetes mellitus and, in addition, presented with multiple chronic illnesses which may have contributed to his susceptibility to invasive disease with a *cnl* strain.

Molecular epidemiology and previous data from serum bactericidal assays (SBA) suggest that some groups of meningococci are more inclined to cause invasive disease than others, with encapsulated strains being more resistant to complement killing than their unencapsulated counterparts [8, 10, 11, 27, 28]. Although the polysaccharide capsule has been shown to be an important virulence determinant, previous SBA data indicate that the ability of the invasive *cnl* ST-192 (cc192) isolates from Burkina Faso to resist complement killing in normal human sera, was comparable to an encapsulated serogroup B strain [8]. The invasive ST-192 (cc192) isolates from Burkina Faso were also determined to be more resistant to complement killing than a carriage ST-53 (cc53) isolate and an invasive ST-845 (cc845) isolate, which had similar resistance profiles to each other and to an unencapsulated serogroup B mutant. Exogenous lipooligosaccharide sialylation significantly increased resistance to complement killing in two invasive *cnl* isolates belonging to ST-198 (cc198), and was partially attributed to their ability to cause invasive disease in apparently healthy patients [9, 10]. However, this mechanism was not identified in the invasive ST-192 (cc192) isolates from Burkina Faso, the carriage ST-53 (cc53) isolate and the invasive ST-845 (cc845) isolate [8].

In agreement with previous genome studies, most putative virulence loci were present in both carriage and invasive *cnl* isolates [29–31]. Although Joseph et al. [32] determined significant associations of mobile genetic elements with invasive meningococci, their contribution to meningococcal virulence is unknown. The ability of *cnl* isolates to cause invasive disease may likely be due to host risk factors, however differences in the virulence potential may also be explained by variation in gene expression. Predisposing factors for one patient with invasive disease that was caused by an ST-192 (cc192) isolate, were unknown. It is likely that this patient may have also presented with underlying disease, however SBA data for the invasive ST-192 (cc192) isolates from Burkina Faso, indicate that these isolates may cause invasive disease in healthy patients. In contrast, cc53 which was previously shown to be sensitive to normal human sera, may require an immunocompromised host to cause IMD, as demonstrated in our study. We did not perform serum bactericidal assays to confirm these findings.

In South Africa, the quadrivalent polysaccharide vaccine and the quadrivalent conjugate vaccine which was recently introduced in 2015, are recommended for individuals with terminal complement deficiencies and may be offered to close contacts of IMD patients following post-exposure chemoprophylaxis. The vaccination status of all three patients as well as the contacts for the first two were unknown, however the mother of the patient with C6 deficiency was administered a single dose of Menactra®, which is ineffective in preventing carriage of *cnl* meningococci which lack a capsule. All 94 *cnl* meningococci including those analyzed in this study lacked the *nadA* locus and the P1.4 antigen; and most isolates expressed fHbp variants which are not targeted by the Bexsero® vaccine (with the exception of isolates that belong to cc198 and cc41/44, which express variant 1 fHbp). The effectiveness of Bexsero® to target *cnl* meningococci in general would therefore be largely reliant on the expression and cross protective potential of NHBA [15]. Although the bivalent fHbp vaccine Trumenba® potentially elicits broad spectrum bactericidal activity against serogroup B strains, its effect on fHbp variants and their level of expression in other serogroups and *cnl* meningococci is unknown [17].

## Conclusion

Invasive meningococcal disease by *cnl* meningococci in South Africa is rare however such strains may have a heightened tendency to cause IMD in an immunocompromised host, potentially coupled with currently unknown non-capsular virulence mechanisms in the meningococcus.

## Methods

### Meningococcal surveillance, 2003–2013

National laboratory-based surveillance for IMD in South Africa was established in 1999 [33] and was enhanced in 2003 through the Group for Enteric, Respiratory and Meningeal Disease Surveillance (GERMS-SA) [34]. Approximately 200 microbiology laboratories from the private and public sector submitted meningococcal isolates and/or clinical specimens together with patient demographic information to the National Institute for Communicable Diseases (NICD) for confirmation and characterization. A case of IMD was defined as the identification of *N. meningitidis* from a normally sterile site specimen by culture, Gram stain and/or antigen detection-latex agglutination result, or a positive PCR result [35, 36]. If a case of IMD was reported ≥21 days after the first episode, it was regarded as a new case.

### Bacterial culture and characterization

At the NICD, *N. meningitidis* identification was confirmed using standard microbiological methods [37]. Minimum inhibitory concentrations for penicillin, chloramphenicol, rifampicin, ciprofloxacin, trimethoprim-sulfamethoxazole, and ceftriaxone were determined using E-test® (bioMérieux, Marcy-l’Étoile, France), and interpreted using Clinical and Laboratory Standards Institute guidelines [38]. Phenotypic serogrouping was performed using capsule-specific antibodies (Remel Biotech Ltd, Dartford, United Kingdom) for detection of serogroups A, B, C, W, X and Y. Real-time PCR detection of *ctrA*, and genogrouping were performed to detect serogroups A (*csaB*), B (*csb*), C (*csc*), W (*csw*), X (*csxB*), and Y (*csy*) [36]. From 2003 through 2013, we identified five IMD isolates which were negative for *ctrA* and six serogroups. *N. meningitidis* identity was reconfirmed using API-NH (bioMérieux, Marcy-l’Étoile, France) and real-time PCR to detect the superoxide dismutase (*sodC*) gene [35]. The five non-groupable isolates were characterized by whole genome sequencing.

### Genome sequencing, assembly and annotation

The Wizard® Genomic DNA Purification Kit (Promega, Madison, USA) was used to extract DNA from suspensions prepared from overnight cultures, according to manufacturer instructions. DNA was quantified using the Qubit® 2.0 fluorometer (Invitrogen, Oregon, USA) and Qubit® dsDNA BR assay kit. Library preparation was performed using the Nextera XT DNA Library Prep Kit (Illumina, California, USA), and sequenced using the Illumina platform. The reads were *de novo* assembled using Velvet (version 1.2.08) combined with the VelvetOptimiser script (version 2.2.4) to a draft level [39, 40]. The minimum output contiguous assembly size was set to 100bp with scaffolding turned off and all other parameters were set as default. No read trimming was performed. The sequence assemblies were uploaded into PubMLST.org/*Neisseria*. Annotation of the genomes was performed using the PubMLST *Neisseria* database, which implements the Bacterial Isolate Genome Sequence (BIGSdb) platform and are publically available [PubMLST: 29306, 29312, 37616, 41860 and 41961] [41]. Additionally, Illumina sequencing was performed for four non-groupable cc192 isolates from Burkina Faso (two carriage and two invasive) at Public Health England, Colindale [PubMLST: 35416, 35417, 35418 and 35419] (Additional file 2: Table S1) [8, 20]. Sequence reads were also deposited in the European Nucleotide Archive (ENA) (http://www.ebi.ac.uk/ena), for the South African [accession: ERR519863, ERR519789, ERR519785, ERR1805704 and ERR1805705] and Burkina Faso isolates [accession: ERR903637, ERR903631, ERR903647 and ERR903634].

### Identification of capsule null isolates

Genome Comparator, a BIGSdb tool, was used to verify PCR negative results for serogroups A, B, C, X, W and Y, and to determine if isolates were serogroups E, H, I, K, L or Z; or harboured a *cnl* allele (PubMLST *Neisseria* database locus identifier: NEIS2743) [2, 6, 41]. Capsule regions A and C were further investigated to confirm the presence of the *cnl* allele using CLC Genomics Workbench version 7.5.1 (CLC bio, Aarhus, Denmark). Additional non-groupable *N. meningitidis* genomes harbouring a *cnl* allele were identified in the PubMLST *Neisseria* database at the time of this analysis, for phylogenomic comparison with the South African isolates (Accessed: 01 July 2016 ). In addition, genes flanking the *cnl* allele, namely, *galE* (NEIS0048) and *tex* (NEIS0059), were compared in all isolates in the PubMLST *Neisseria* database.

### Strain typing of capsule null isolates

Species identity was confirmed *in silico* by the presence of *sodC* (NEIS1339) and analysis of a 413bp fragment of the 50S ribosomal protein L6 (*rplF,* NEIS0147) [42]. Multilocus sequence type (ST) and peptide typing fragments for porin A (PorA) variable regions (VR) 1 and 2, ferric enterochelin receptor (FetA) VR, factor H-binding protein (fHbp), neisserial adhesin A (NadA) and neisserial heparin-binding antigen (NHBA), were identified from the whole genome data [43–46].

### Phylogenomic comparison of capsule null meningococci

Genome Comparator was used to construct phylogenetic networks to assess the relationships between the South African *cnl* isolates and additional *cnl* genomes. Isolates were compared using seven MLST genes, 53 rMLST genes and 1605 core genes [cgMLST scheme v1.0] [47–49]. The distance matrices were visualized as Neighbor-net phylogenies and annotated using SplitsTree version 4.13.1 [50]. The degree of relatedness between isolates was quantified by calculating the mean number of differing core loci between isolates and statistical significance was determined using the Fisher’s exact test (*p* < 0.05). Loci which were absent in at least one isolate or incomplete as a result of being situated at the end of a contig, were excluded from analysis. Functional annotations for variable core loci were determined using the PubMLST *Neisseria* database.

### Identification of genetic markers for potential differentiation of carriage and invasive capsule null meningococci

The Genome Comparator tool was used to examine 117 previously defined putative virulence loci, in 51 carriage and 14 invasive capsule null isolates with known epidemiology (Additional file 4: Table S3) [30]. The ST-192 (cc192) isolates from Malawi were not included in the analysis as genome data were not available on the PubMLST *Neisseria* database at the time of analysis (22 December 2016). A mutually exclusive gene or allele was defined as being present in all isolates in one group (carriage or invasive) and absent in the other.

### Transmission electron microscopy

The South African isolates were visualized using a previously described TEM method that was adapted for Gram-negative bacteria by substituting ruthenium red with a 0.5% alcian blue pyridine variant (pH 7.2) [51]. Ultrathin sections were viewed using a 120 kV BioTwin Spirit transmission electron microscope (FEI Company, Oregon, USA). American Type Culture Collection (ATCC®) isolate M-603, a *N. meningitidis* serogroup W strain (ATCC-35559™) was used as an encapsulated control. NCDC A7515, a *N. lactamica* strain (ATCC-23970™) was used as an unencapsulated control.

## Additional files

Additional file 1: Figure S1. Nucleotide sequences of capsule null locus (*cnl*) alleles identified in invasive and carried *Neisseria meningitidis* isolates analyzed in this study (*n* = 93), PubMLST *Neisseria* database locus identifier: NEIS2743. (TIFF 570 kb)
Additional file 2: Table S1. Epidemiological information, molecular characterization and genome characteristics of all *Neisseria meningitidis* isolates analyzed in this study (*n* = 94). (XLSX 18 kb)
Additional file 3: Table S2. Variable core loci (*n* = 2) identified in clonal complex 192 capsule null *Neisseria meningitidis* isolates 41860 and 41961, obtained from a patient with recurrent invasive meningococcal disease in 2011 and 2012, respectively. (XLSX 9 kb)
Additional file 4: Table S3. Putative virulence loci (*n* = 117) analyzed in 51 carriage and 14 invasive capsule null meningococci, adapted from Schoen et al. [30]. (XLSX 13 kb)

## Abbreviations

ATCC: American Type Culture Collection
base-pair: bp
BIGSdb: Bacterial Isolate Genome Sequence database
C5: Fifth complement component
C6: Sixth complement component
C9: ninth complement component
cc: clonal complex
cgMLST: core genome MLST
*cnl*: capsule null locus
COPD: Chronic obstructive pulmonary disease
*cps*: capsular polysaccharide synthesis
CSF: Cerebrospinal fluid
F: FetA
FetA: Ferric enterochelin receptor
fHbp: factor H-binding protein
GERMS-SA: Group for Enteric, Respiratory and Meningeal Disease Surveillance
HIV: Human immunodeficiency virus
IMD: Invasive meningococcal disease
MIC: Minimum inhibitory concentration
MLST: Multilocus sequence typing
*N. meningitidis*: *Neisseria meningitidis*
NadA: Neisserial adhesin A
NG: Non-groupable
NHBA: Neisserial heparin-binding antigen
NICD: National Institute for Communicable Diseases
P1: PorA
PCR: Polymerase chain reaction
PorA: Porin A
R: Resistant
rMLST: ribosomal multilocus sequence typing
S: Susceptible
SA: South Africa
ST: Sequence type
TEM: Transmission electron microscopy
UK: United Kingdom
VR: Variable region
Δ: Gene deletion

## References

[1] Kahler CM, Martin LE, Shih GC, Rahman MM, Carlson RW, Stephens DS (1998). The (alpha2-- > 8)-linked polysialic acid capsule and lipooligosaccharide structure both contribute to the ability of serogroup B *Neisseria meningitidis* to resist the bactericidal activity of normal human serum. Infect Immun.

[2] Harrison OB, Claus H, Jiang Y (2013). Description and nomenclature of *Neisseria meningitidis* capsule locus. Emerg Infect Dis.

[3] Frosch M, Weisgerber C, Meyer TF (1989). Molecular characterization and expression in *Escherichia coli* of the gene complex encoding the polysaccharide capsule of *Neisseria meningitidis* group B. Proc Natl Acad Sci U S A.

[4] Hammerschmidt S, Birkholz C, Zahringer U (1994). Contribution of genes from the capsule gene complex (cps) to lipooligosaccharide biosynthesis and serum resistance in *Neisseria meningitidis*. Mol Microbiol.

[5] Petering H, Hammerschmidt S, Frosch M, van Putten JPM, Ison CA, Robertson BD (1996). Genes associated with meningococcal capsule complex are also found in *Neisseria gonorrhoeae*. J Bacteriol.

[6] Claus H, Maiden MCJ, Maag R, Frosch M, Vogel U (2002). Many carried meningococci lack the genes required for capsule synthesis and transport. Microbiology.

[7] Dolan-Livengood JM, Miller YK, Martin LE, Urwin R, Stephens DS (2003). Genetic basis for nongroupable *Neisseria meningitidis*. J Infect Dis.

[8] Findlow H, Vogel U, Mueller JE (2007). Three cases of invasive meningococcal disease caused by a capsule null locus strain circulating among healthy carriers in Burkina Faso. J Infect Dis.

[9] Hoang LM, Thomas E, Tyler S (2005). Rapid and fatal meningococcal disease due to a strain of *Neisseria meningitidis* containing the capsule null locus. Clin Infect Dis.

[10] Johswich KO, Zhou J, Law DK (2012). Invasive potential of nonencapsulated disease isolates of *Neisseria meningitidis*. Infect Immun.

[11] Vogel U, Claus H, von Muller L, Bunjes D, Elias J, Frosch M (2004). Bacteremia in an immunocompromised patient caused by a commensal *Neisseria meningitidis* strain harboring the capsule null locus (cnl). J Clin Microbiol.

[12] Xu Z, Zhu B, Xu L, Gao Y, Shao Z (2015). First case of *Neisseria meningitidis* capsule null locus infection in China. Infect Dis (Lond).

[13] Claus H, Maiden MCJ, Wilson DJ (2005). Genetic analysis of meningococci carried by children and young adults. J Infect Dis.

[14] Ibarz-Pavon AB, Maclennan J, Andrews NJ (2011). Changes in serogroup and genotype prevalence among carried meningococci in the United Kingdom during vaccine implementation. J Infect Dis.

[15] Claus H, Jordens MS, Kriz P (2012). Capsule null locus meningococci: typing of antigens used in an investigational multicomponent meningococcus serogroup B vaccine. Vaccine.

[16] Giuliani MM, Adu-Bobie J, Comanducci M (2006). A universal vaccine for serogroup B meningococcus. Proc Natl Acad Sci U S A.

[17] Jiang HQ, Hoiseth SK, Harris SL (2010). Broad vaccine coverage predicted for a bivalent recombinant factor H binding protein based vaccine to prevent serogroup B meningococcal disease. Vaccine.

[18] Kristiansen PA, Ba AK, Ouedraogo AS (2014). Persistent low carriage of serogroup A *Neisseria meningitidis* two years after mass vaccination with the meningococcal conjugate vaccine, MenAfriVac. BMC Infect Dis.

[19] Leimkugel J, Hodgson A, Forgor AA (2007). Clonal waves of *Neisseria* colonisation and disease in the African meningitis belt: eight-year longitudinal study in northern Ghana. PLoS Med.

[20] Mueller JE, Sangare L, Njanpop-Lafourcade BM (2007). Molecular characteristics and epidemiology of meningococcal carriage, Burkina Faso, 2003. Emerg Infect Dis.

[21] Fijen CA, Kuijper EJ, Tjia HG, Daha MR, Dankert J (1994). Complement deficiency predisposes for meningitis due to nongroupable meningococci and *Neisseria*-related bacteria. Clin Infect Dis.

[22] Hummell DS, Mocca LF, Frasch CE (1987). Meningitis caused by a nonencapsulated strain of *Neisseria meningitidis* in twin infants with a C6 deficiency. J Infect Dis.

[23] Kemp AS, Vernon J, Muller-Eberhard HJ, Bau DC (1985). Complement C8 deficiency with recurrent meningococcemia: examination of meningococcal opsonization. Aust Paediatr J.

[24] Orren A, Owen EP, Henderson HE (2012). Complete deficiency of the sixth complement component (C6Q0), susceptibility to *Neisseria meningitidis* infections and analysis of the frequencies of C6Q0 gene defects in South Africans. Clin Exp Immunol.

[25] Owen EP, Wurzner R, Leisegang F (2015). A complement C5 gene mutation, c.754G > A:p.A252T, is common in the Western Cape, South Africa and found to be homozygous in seven percent of Black African meningococcal disease cases. Mol Immunol.

[26] Zoppi M, Weiss M, Nydegger UE, Hess T, Späth PJ (1990). Recurrent meningitis in a patient with congenital deficiency of the C9 component of complement. First case of C9 deficiency in Europe. Arch Intern Med.

[27] Caugant DA, Maiden MCJ (2009). Meningococcal carriage and disease—population biology and evolution. Vaccine.

[28] Maiden MCJ (2006). Multilocus sequence typing of bacteria. Annu Rev Microbiol.

[29] Marri PR, Paniscus M, Weyand NJ (2010). Genome sequencing reveals widespread virulence gene exchange among human *Neisseria* species. PLoS One.

[30] Schoen C, Blom J, Claus H (2008). Whole-genome comparison of disease and carriage strains provides insights into virulence evolution in *Neisseria meningitidis*. Proc Natl Acad Sci U S A.

[31] Snyder LA, Saunders NJ (2006). The majority of genes in the pathogenic *Neisseria* species are present in non-pathogenic *Neisseria lactamica*, including those designated as ‘virulence genes’. BMC Genomics.

[32] Joseph B, Schwarz RF, Linke B (2011). Virulence evolution of the human pathogen *Neisseria meningitidis* by recombination in the core and accessory genome. PLoS One.

[33] Huebner RE, Klugman KP, Matai U, Eggers R, Hussey G (1999). Laboratory surveillance for *Haemophilus influenzae* type B meningococcal, and pneumococcal disease. *Haemophilus* Surveillance Working Group. S Afr Med J.

[34] von Gottberg A, du Plessis M, Cohen C (2008). Emergence of endemic serogroup W135 meningococcal disease associated with a high mortality rate in South Africa. Clin Infect Dis.

[35] Dolan Thomas J, Hatcher CP, Satterfield DA (2011). *sodC*-based real-time PCR for detection of *Neisseria meningitidis*. PLoS One.

[36] Wang X, Theodore MJ, Mair R (2012). Clinical validation of multiplex real-time PCR assays for detection of bacterial meningitis pathogens. J Clin Microbiol.

[37] Winn W, Allen S, Janda W (2006). Koneman’s color atlas and textbook of diagnostic microbiology.

[38] Clinical and Laboratory Standards Institute (2014). M100-S24 Performance standards for antimicrobial susceptibility testing; twenty-fourth informational supplement.

[39] Zerbino DR (2010). Using the Velvet *de novo* assembler for short-read sequencing technologies. Curr Protoc Bioinformatics.

[40] Zerbino DR, Birney E (2008). Velvet: algorithms for *de novo* short read assembly using de Bruijn graphs. Genome Res.

[41] Jolley KA, Maiden MCJ (2010). BIGSdb: Scalable analysis of bacterial genome variation at the population level. BMC Bioinformatics.

[42] Bennett JS, Watkins ER, Jolley KA, Harrison OB, Maiden MCJ (2014). Identifying *Neisseria* species by use of the 50S ribosomal protein L6 (rplF) gene. J Clin Microbiol.

[43] Brehony C, Wilson DJ, Maiden MCJ (2009). Variation of the factor H-binding protein of *Neisseria meningitidis*. Microbiology.

[44] Comanducci M, Bambini S, Brunelli B (2002). NadA, a novel vaccine candidate of *Neisseria meningitidis*. J Exp Med.

[45] Jolley KA, Brehony C, Maiden MCJ (2007). Molecular typing of meningococci: recommendations for target choice and nomenclature. FEMS Microbiol Rev.

[46] Serruto D, Spadafina T, Ciucchi L (2010). *Neisseria meningitidis* GNA2132, a heparin-binding protein that induces protective immunity in humans. Proc Natl Acad Sci U S A.

[47] Bratcher HB, Corton C, Jolley KA, Parkhill J, Maiden MCJ (2014). A gene-by-gene population genomics platform: de novo assembly, annotation and genealogical analysis of 108 representative *Neisseria meningitidis* genomes. BMC Genomics.

[48] Jolley KA, Bliss CM, Bennett JS (2012). Ribosomal multilocus sequence typing: universal characterization of bacteria from domain to strain. Microbiology.

[49] Maiden MCJ, Bygraves JA, Feil E (1998). Multilocus sequence typing: a portable approach to the identification of clones within populations of pathogenic microorganisms. Proc Natl Acad Sci U S A.

[50] Huson DH (1998). SplitsTree: analyzing and visualizing evolutionary data. Bioinformatics.

[51] Hammerschmidt S, Wolff S, Hocke A, Rosseau S, Muller E, Rohde M (2005). Illustration of pneumococcal polysaccharide capsule during adherence and invasion of epithelial cells. Infect Immun.

