# Supplementary figures and images for: Molecular characterization of invasive capsule null Neisseria meningitidis in South Africa
Source: BMC Microbiol. 2017 Feb 21;17:40. doi: 10.1186/s12866-017-0942-5 (PMC5320719; doi:10.1186/s12866-017-0942-5)

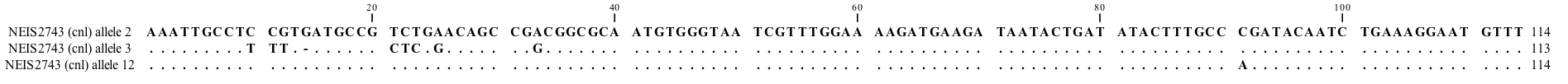

Supplement: Additional file 1: Figure S1. — Nucleotide sequences of capsule null locus (cnl) alleles identified in invasive and carried Neisseria meningitidis isolates analyzed in this study (n = 93), PubMLST Neisseria database locus identifier: NEIS2743. (TIFF 570 kb) [file 12866_2017_942_MOESM1_ESM.tiff]
